# PKD1 deficiency induces Bronchiectasis in a porcine ADPKD model

**DOI:** 10.1186/s12931-022-02214-3

**Published:** 2022-10-29

**Authors:** Runming Wang, Wenya Li, Haiting Dai, Mingli Zhu, Lingyu Li, Guohui Si, Yilina Bai, Hanyu Wu, Xiaoxiang Hu, Yiming Xing

**Affiliations:** State Key Laboratory for Agrobiotechnology, College of Biological Sciences, China Agricultural University, Beijing, P.R. China

**Keywords:** ADPKD, *PKD1*, Pig, Bronchiectasis, E-cadherin

## Abstract

**Background:**

Autosomal dominant polycystic kidney disease (ADPKD) is a prevalent genetic disorder, mainly characterized by the development of renal cysts, as well as various extrarenal manifestations. Previous studies have shown that ADPKD is related to bronchiectasis, while its pathogenic mechanism is unclear. In previous studies, we have generated the *PKD1*^*+/−*^ pigs to simulate the progression of cyst formation and physiological alterations similar to those seen in ADPKD patients.

**Methods:**

Phenotypic changes to airway epithelial cell and mesenchymal cell in *PKD1*^*+/−*^ pigs were assessed by histological analysis. The molecular mechanisms driving these processes were investigated by using *PKD1*^*+/−*^ pig lungs, human mesenchymal cells, and generating *PKD1* deficient human epithelial cells.

**Results:**

We identified bronchiectasis in *PKD1*^*+/−*^ pigs, which is consistent with the clinical symptoms in ADPKD patients. The deficiency of *PKD1* suppressed E-cadherin expression in the airway epithelial barrier, which aggravated invasion and leaded to a perpetuated inflammatory response. During this process, extracellular matrix (ECM) components were altered, which contributed to airway smooth muscle cell phenotype switch from a contractile phenotype to a proliferative phenotype. The effects on smooth muscle cells resulted in airway remodeling and establishment of bronchiectasis.

**Conclusion:**

To our knowledge, the *PKD1*^*+/−*^ pig provides the first model recapitulating the pathogenesis of bronchiectasis in ADPKD. The role of *PKD1* in airway epithelial suggests a potential target for development of new strategies for the diagnosis and treatment of bronchiectasis.

**Supplementary Information:**

The online version contains supplementary material available at 10.1186/s12931-022-02214-3.

## Introduction

Bronchiectasis is a chronic disease of persistent lung inflammation and recurrent infections and is defined by irreversible bronchial dilation due to various etiologies [1]. It is the result of a complex interplay between host immunity, pathogen, and environment [2]. The dysfunctional host immunity to viral, bacterial, or fungal species leads to chronic inflammation, lung remodeling, and damaged lung epithelium associated with recurrent infection and impaired pathogen clearance. Bronchiectasis is a pathological endpoint consisting of abnormal dilatation of the bronchi and bronchioles. The extent of bronchiectasis can range from focal disease, limited to one segment or lobe, to diffuse disease, involving both lungs in all lobes [3]. Patients with severe bronchiectasis associate with chronic obstructive pulmonary disease (COPD), wheezing, pulmonary hypertension, chronic respiratory failure, and life-threatening haemoptysis [4].

The list of conditions known to cause bronchiectasis is long but most share common features. The pathophysiologic pathway generally starts with impaired mucociliary clearance which promotes establishment of chronic infections. Cilia are classified as motile or primary. Motile cilia are well-differentiated epithelial cells, whereas primary cilia are non-motile and implicated in the regulation of fundamental processes in development. Analysis of airway epithelial cell differentiation showed that motile ciliated cells originate from primary ciliated cells [5]. The expression of polycystin-1 (PC1) has been detected in both primary and motile cilia in the airways and it is markedly increased during differentiation, suggesting that PC1 regulates cilia differentiation in the lungs [5]. PC1 is a large membrane-bound protein expressed on the cilia and acts as a mechanosensor for extracellular signals. Its deficiency is associated with decreased intracellular calcium concentrations, resulting in cystogenesis through activation of proliferation and secretion pathways [6]. PC1 is encoded by polycystic kidney disease 1 (*PKD1*), and *PKD1* mutations causes Autosomal dominant polycystic kidney disease (ADPKD) [7]. ADPKD is an inherited genetic disorder that primary ciliary abnormality in renal epithelial cells results in cellular hypertrophy, hyperplasia and cyst formation [8]. Since patients with ADPKD also have bronchiectasis, it suggests that there is a link between cilia, cellular signaling processes, and bronchiectasis [9]. Thus, ADPKD is a ciliopathy rather than a renal disease. Molecular research is mandatory for developing new therapeutic tools [10] and to better understand disease history [11]. During the process of cyst generation, many signaling pathways are involved, including the MAPK, mTOR, Wnt, STAT3, Smyd2 and TGFβ pathways [6, 8]. Nevertheless, the role of PC1 in contributing the increased prevalence of bronchiectasis in ADPKD patients remains undiscovered.

Identifying the etiology of bronchiectasis is of great significance to guide treatment, and discovering the mechanisms underlining the pathogenesis of bronchiectasis is largely relied on animal models. The apical lobe ligation was once used to generate bronchiectasis in Wistar outbred rats in 1989 [12]. Although the cellular immune response was present as in human patients, this model did not simulate idiopathic bronchiectasis. In 2014, airway cilia were deleted by knockout of IFT88 in mice, leading to bronchiectasis [13]. Yet, there were no significant changes in inflammation response and respiratory mucus secretions, which is contrast to patients with bronchiectasis. Nowadays, the development of new therapies for bronchiectasis has many challenges and is greatly limited by the lack of an adequate animal model of bronchiectasis [14]. Therefore, an ideal animal model for bronchiectasis is necessary to study and develop new therapies.

In terms of body size, anatomy, metabolism, physiology and pathophysiological responses, pig is an attractive model for biomedical research [15]. In previous studies, we have generated the *PKD1*^*+/−*^ pigs to simulate the progression of cyst formation and physiological alterations similar to those seen in ADPKD patients [16]. The aim of this study was to investigate the mechanism of bronchiectasis in ADPKD caused by *PKD1* deficiency. By analyzing the *PKD1* deficient pig lungs, we identified bronchiectasis and discovered that expression of E-cadherin was decreased in airway epithelial cells, which in turn promoted cellular barrier damage and an aggravated inflammatory response. The alteration of extracellular matrix (ECM) components in *PKD1*^*+/−*^ epithelial lead to an airway smooth muscle cell phenotype switch and resulted in airway remodeling and bronchiectasis formation. The *PKD1*^*+/−*^ pig model provided a valuable tool to discover the mechanism of the progression of bronchiectasis, and the role of PKD1 in lung epithelial suggested a potential target to develop new strategy for the treatment of bronchiectasis.

## Materials and methods

### Animal models

The *PKD1*^*+/−*^ Chinese experimental mini-pigs (CEMP) were generated in earlier studies [16]. The pig species (CEMP) was bred by China Agricultural University. All animal studies were performed according to the Chinese Animal Welfare Act, and conducted according to protocols approved by China Agricultural University (Number AW10201202-3-1).

### Cell lines and cell culture

The human lung epithelial cell line (H1299) and the human fetal lung fibroblast cell line (MRC5) were purchased from China Center for Type Culture Collection, China. The cells were cultured in RPMI-1640 or DMEM medium supplemented with 10% fetal bovine serum (Gibco, USA), 100U/mL penicillin and 100 µg/mL streptomycin (Gibco, USA) at 37 °C in humidified air containing 5% CO_2_. Collagen I (Sigma-Aldrich, USA) was dissolved in 0.1mM acetic acid to obtain a 50 µg/ml solution as the stock solution. The surfaces of six-well cell culture plates were coated with collagen I (0.5ml/well) overnight and dried at room temperature. MRC5 cells were seeded in collagen coated plates (75,000 cells/well) and the medium was refreshed every 48 h. Upon reaching confluence, the cells were collected for analyses.

### Generation of PKD1^KD^ cells

H1299 cells were used to generate *PKD1*^*KD*^ cells and the CRISPR-Cas13d system was used to generate mutations in the *PKD1* gene. Plasmids were gifts from Dr. Xingxu Huang at the ShanghaiTech University. Vectors expressing Cas13d and sgRNA were co-transfected into H1299 cells. The human *PKD1*-sgRNA sequence was designed as GATATAGCCAAAGGGAAAGGGATTGGAG.

### Contrast enhanced computed tomography

Pigs were anesthetized by 3 mg/kg ketamine intravenously and CT scanning was performed in the Zhuozhou Chinese Medical Hospital with a multi-splice spiral CT instrument (Siemens), with the following parameters: 120 kV, 500ms, and 1.5 mm splice thickness. The software Syngo Fastview was used to perform the 3D reconstruction.

### RNA isolation and real-time PCR

Total RNA was extracted from lungs or cells by a Qiagen RNeasy Mini Kit (QIAGEN, Duesseldorf, Germany) according to the manufacturer’s protocol. RNA was reverse-transcribed into cDNAs using M-MLV Reverse Transcriptase Kit (Promega, USA). Real-time quantification PCR analysis was performed by using the ABI prism 7300 system. *GAPDH* was used as an internal control gene. All primers were designed by Primer3web (version 4.1.0) and listed in Supplemental Table 1.

### Western blot analysis

Total proteins were extracted by RIPA lysis buffer containing 1mM PMSF (Beyotime, China) and PhosSTOP EASY pack (Roche, Switzerland). Equal amounts of protein were separated by SDS/PAGE gels, transferred to Immobilon-P transfer membranes, and hybridized to an appropriate primary antibody and HRP-conjugated secondary antibody for subsequent detection by enhanced chemiluminescence (Millipore). The intensity of bands was analyzed by ImageJ software. Primary antibodies used in this study are listed in Supplemental Table 2.

### Histological analysis

Lung tissues were fixed in 10% neutral formalin, embedded and processed into serial sections (4 μm) using standard procedures. Sections were incubated with specific primary antibodies respectively. Haematoxylin-eosin (H&E) staining was performed for morphological examination. Size of the scale bar for each experiment is indicated in the figure legends. Primary antibodies used in this study are listed in Supplemental Table 2.

### Statistical analysis

Statistical analyses were performed with the SPSS 16.0 software. Statistical significance between two groups was determined using an unpaired two-tailed Student’s *t*-test. Data are presented as mean ± SD (standard deviation) as indicated in the figure legends. *P*-values were considered statistically significant at *P* < 0.05.

## Results

### PKD1^+/−^ pigs display bronchiectasis

**Fig. 1:**
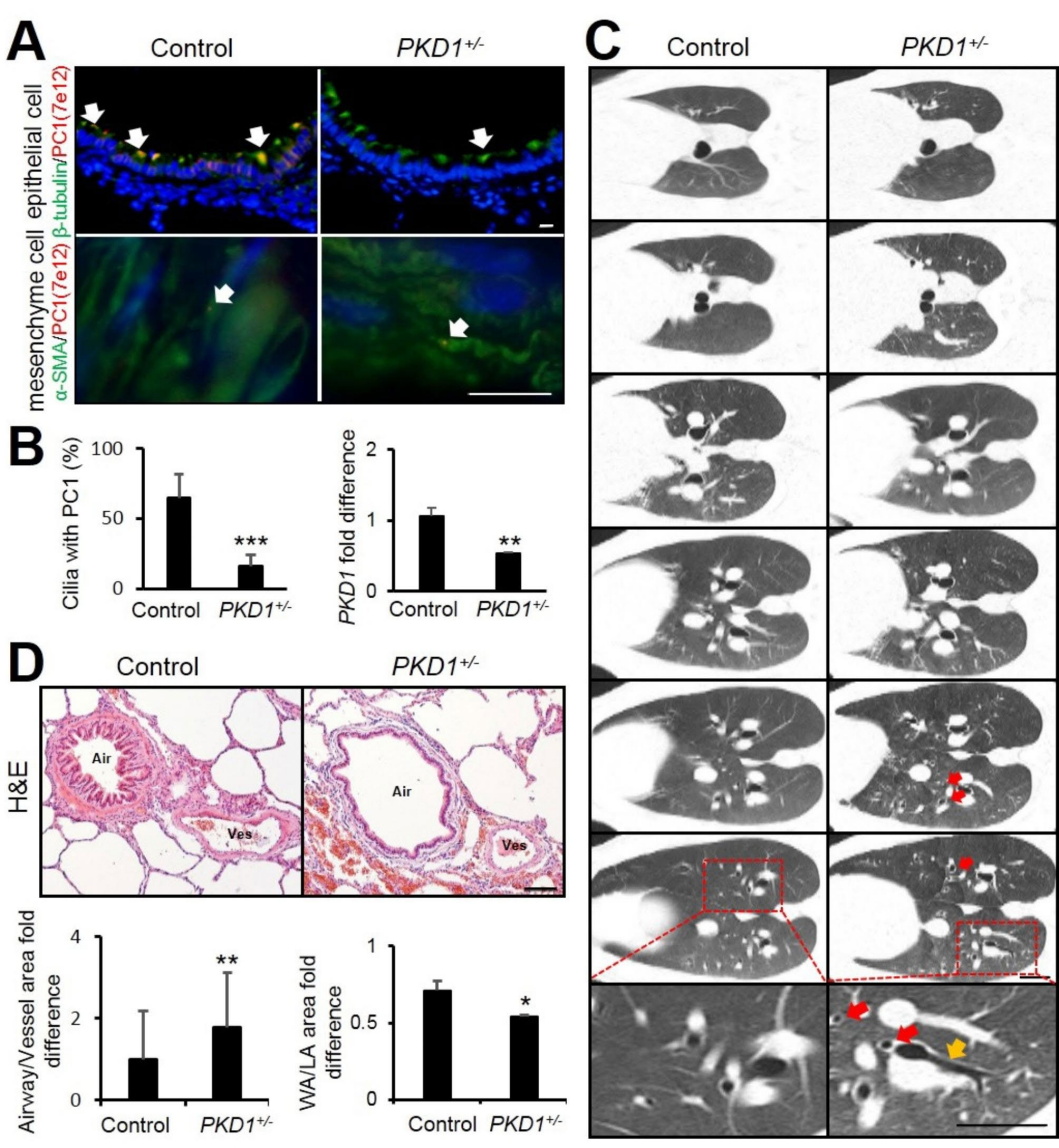
***PKD1***^***+/−***^**pigs displayed bronchiectasis. (A)** Immunofluorescence staining of β-tubulin, α-SMA and PC1 in 2-year-old pig airways. Scale bar: 10 μm. (**B)** Quantification of airways cilia with PC1 expression and real-time PCR analyses of *PKD1* expression in 2-year-old pigs. n = 3. (**C)** Detection of bronchiectasis by CT scan in 4-year-old pigs. Scale bar: 25 mm. Signet-ring sign, which meant the airway (hollow circular) was larger than the blood vessel (solid circular), was shown by red arrows and tram-track sign was used to show non-tapering of the bronchi (yellow arrows). (**D)** H&E staining of pig airways, the area ratio of airway to adjacent artery and the area ratio of airway wall to lumen were measured in 2-year-old pigs, more than 30 slices were quantified for each group. Scale bar: 100 μm. The bars represent the mean ± SD; **P < 0.01, ***P < 0.001

Previous studies have shown that PC1 is expressed in airway cilia cells, as well as airway and vascular smooth muscle in humans and rodents [17, 18]. To identify whether PC1 was also expressed in pig lungs, an immunofluorescence assay lungs from 2-year old pigs was performed. Anti-β-tubulin was used to detect cilia protein, whereas α-smooth muscle actin (α-SMA) was used as a marker of smooth muscle cells. The co-localization of PC1 and β-tubulin showed that *PKD1* was mainly expressed in airway ciliated cells in the pig lungs. The PC1 expression was significantly decreased in *PKD1*^*+/−*^ pig airways cilia (Fig. 1 A&B). Interestingly, the ablation of PC1 in smooth muscle cells was hardly detectable in *PKD1*^*+/−*^ pig lungs (Fig. 1 A). FOXJ1 is a forkhead-box transcription factor expressed in motile cilia and shown to be required for cilia formation by gene deletion in a mouse model [19]. In the *PKD1*^*+/−*^ pig lungs, no significant changes were observed in the expression of *FOXJ1* (Supplemental Fig. 1), suggesting that the PKD1 deficiency did not affect airway ciliated cell formation.

ADPKD patients show an increased prevalence of bronchiectasis [20]. In previous studies, we have generated an ADPKD pig model by knocking out *PKD1*. The *PKD1*^*+/−*^ pigs showed cysts formation and pathological patterns in their kidneys similar to those in ADPKD patients. Thus, in the current study, we investigated whether *PKD1*^*+/−*^ pigs also suffered from bronchiectasis. Although not all the airways were affected, by CT scanning, bronchiectatic features, “tram line sign” and “signet ring sign”, were observed in *PKD1*^*+/−*^ pig lungs (Fig. 1 C). Since the airway-to-artery diameter is relatively consistent along the respiratory tree, the area ratio of airway to adjacent artery was calculated to determine whether the airways were enlarged in *PKD1*^*+/−*^ pig lungs. Compared to control lungs, the H&E staining results showed that the area ratio of airway to adjacent artery was significantly increased whereas the value of wall area to lumen was decreased in *PKD1*^*+/−*^ lungs (Fig. 1 C), indicating that bronchiectasis occurred in the *PKD1*^*+/−*^ pigs.

### PKD1^+/−^ pig lungs exhibit enhanced inflammation and mucus secretion

**Fig. 2:**
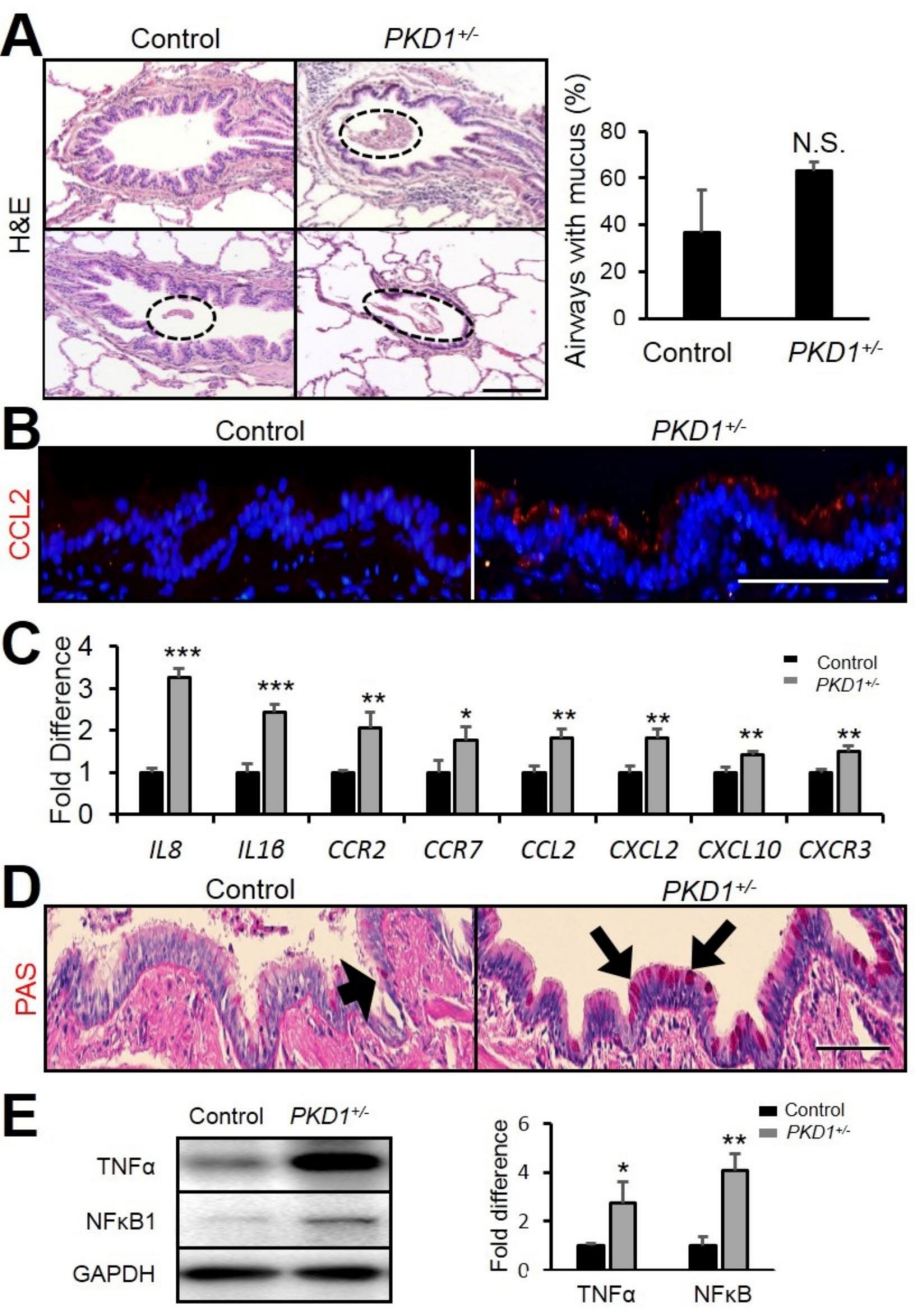
***PKD1*deficiency promoted inflammation and mucus retention in pig lungs. (A)** H&E staining of 2-year-old Control and *PKD1*^*+/−*^ pig airways and quantification of airways with mucus in 2-year-old pigs. n = 3. Scale bar: 100 μm. **(B)** Immunofluorescence staining of CCL2 in 2 years old pig lungs. Scale bar: 100 μm. **(C)** Real-time PCR analysis of inflammation related genes in 2-year-old Control and *PKD1*^*+/−*^ pig lungs. n = 3. **(D)** PAS staining of 2-years-old pig lungs. Scale bar: 100 μm. **(E)** Representative Western blot analyses of TNFα and NFκB1 and their quantifications. n = 3. The bars represent the mean ± SD; N.S. means no significant; *P < 0.05; **P < 0.01; ***P < 0.001.

Patients with bronchiectasis usually show an elevated inflammatory response in the lungs, which in turn exacerbates airway enlargement [21]. By H&E staining, mucus retention and inflammation in the airways were observed in the pig lungs (Fig. 2 A). Although, as compared to controls the number of airways with mucus was not altered, the mucus plugging was much more pronounced in the airways of the *PKD1*^*+/−*^ pigs, suggesting that inflammation was enhanced in the *PKD1*^*+/−*^ airways. Chemokine (C-C motif) ligand 2 (CCL2) is known to be involved in the pathogenesis of inflammatory diseases [22]. By immunofluorescent staining, the expression of CCL2 was shown to be significantly increased in the *PKD1*^*+/−*^ airway epithelial cells (Fig. 2B). Interleukins are critical immune modulators and are important for leucocyte communication [23], whereas chemokines attract white blood cells to sites of infection [24]. By real-time PCR analysis, it showed that the expression of interleukins and chemokines were strongly upregulated in the *PKD1*^*+/−*^ lungs (Fig. 2 C). By Periodic Acid Schiff (PAS) staining, more PAS positive cells were detected in the *PKD1*^*+/−*^ airways comparing with the control airways (Fig. 2D), which indicated that goblet cell metaplasia occurred in the *PKD1*^*+/−*^ airway epithelium. TNFα and NFκB are also related to the inflammatory response [25]. And Western blot results showed that TNFα and NFκB1 levels were increased (Fig. 2E). Thus, the deficiency of *PKD1* in pig lung ciliated cells enhanced inflammation and mucus retention, which is similar to the clinical symptoms in bronchiectasis patients [1].

### PKD1 deficiency in pig lung leads to epithelial barrier dysfunction

**Fig. 3:**
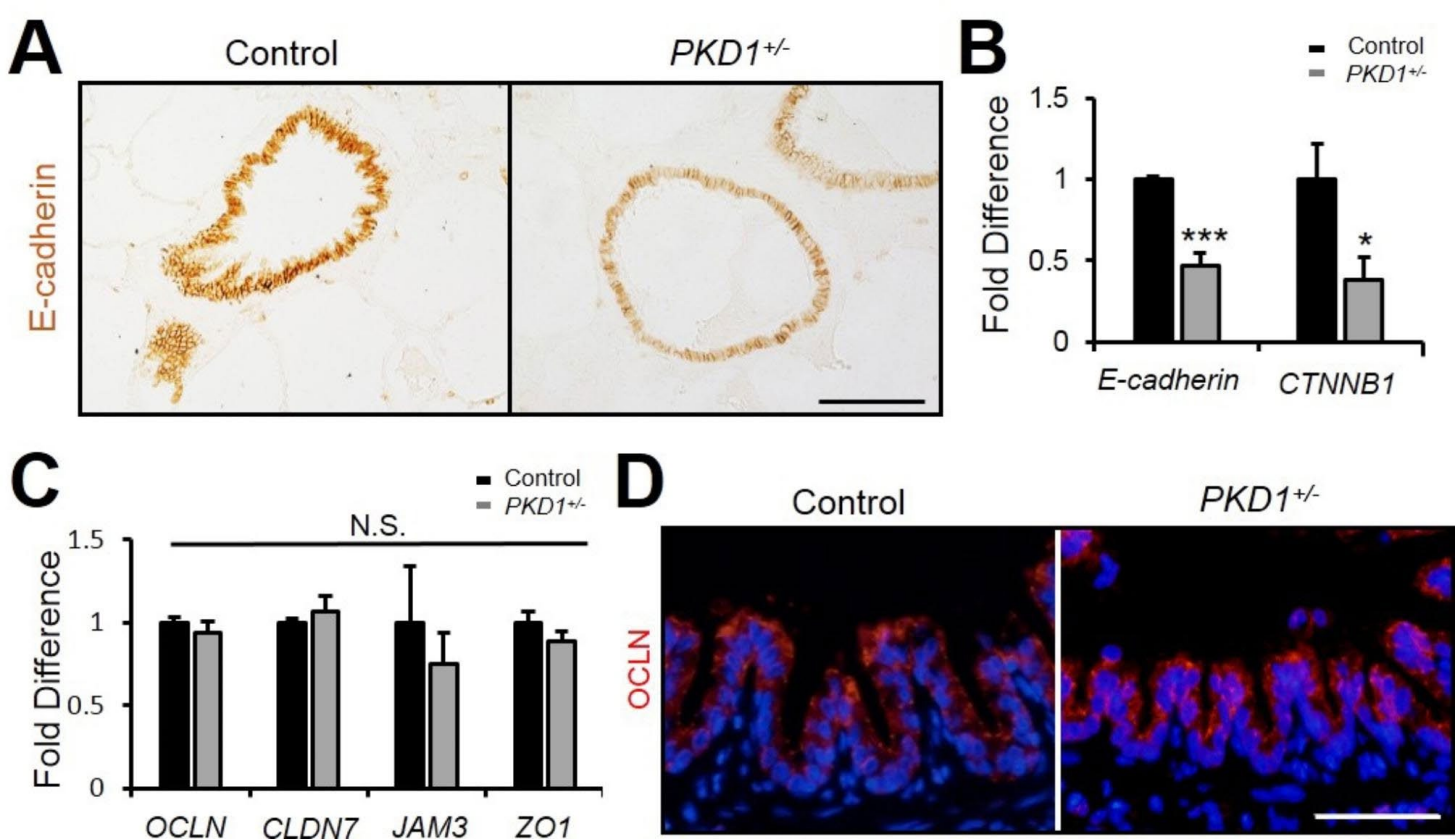
***PKD1*deficiency caused airway epithelial barrier dysfunction. (A)** Immunohistochemical staining of E-cadherin in 2-year-old pig airways. Scale bar: 100 μm. **(B)** Real-time PCR analyses of *E-cadherin* and *CTNNB1* in 2-year-old *PKD1*^*+/−*^ pig lungs. n = 3. **(C)** Real-time PCR analyses of genes related to tight junctions in 2-year-old *PKD1*^*+/−*^ pig lungs. n = 3. **(D)** Immunofluorescence staining of occludin in 2-year-old pig airways. Scale bar: 50 μm. The bars represent the mean ± SD; N.S. means no significant; **P < 0.01; ***P < 0.001.

The epithelial lining forms the first barrier to environmental insults and plays important roles in the innate immune system [26]. Since the airway epithelium junctions, including adherens junctions (AJs) and tight junctions (TJs), form the epithelial barrier, deficiency of epithelial junctions will predispose to infections and increase the morbidity of airway diseases, such as asthma, cystic fibrosis (CF) and chronic obstructive pulmonary disease (COPD) [27]. AJs consist of the transmembrane protein E-cadherin, and the intracellular components, p120-catenin, β-catenin and α-catenin [28]. By immunohistochemical staining and real-time PCR analyses, we showed that E-cadherin and β-catenin (encoded by *CTNNB1*) were downregulated in the *PKD1*^*+/−*^ lungs (Fig. 3 A & B). Transmembrane protein occludin (OCLN), CLDN7, JAM3 and ZO1 are major components of TJs [26]. By real-time PCR analysis, the expressions of their encoding genes were not changed (Fig. 3 C). The immunofluorescent staining result confirmed that the expression of OCLN was not altered in the *PKD1*^*+/−*^ lungs (Fig. 3D). The above results indicate that the *PKD1* deficiency in *PKD1*^*+/−*^ lungs affects airway AJs but not TJs.

### Absence of PKD1 in lung cells affects E-cadherin but not p38MAPK.

**Fig. 4:**
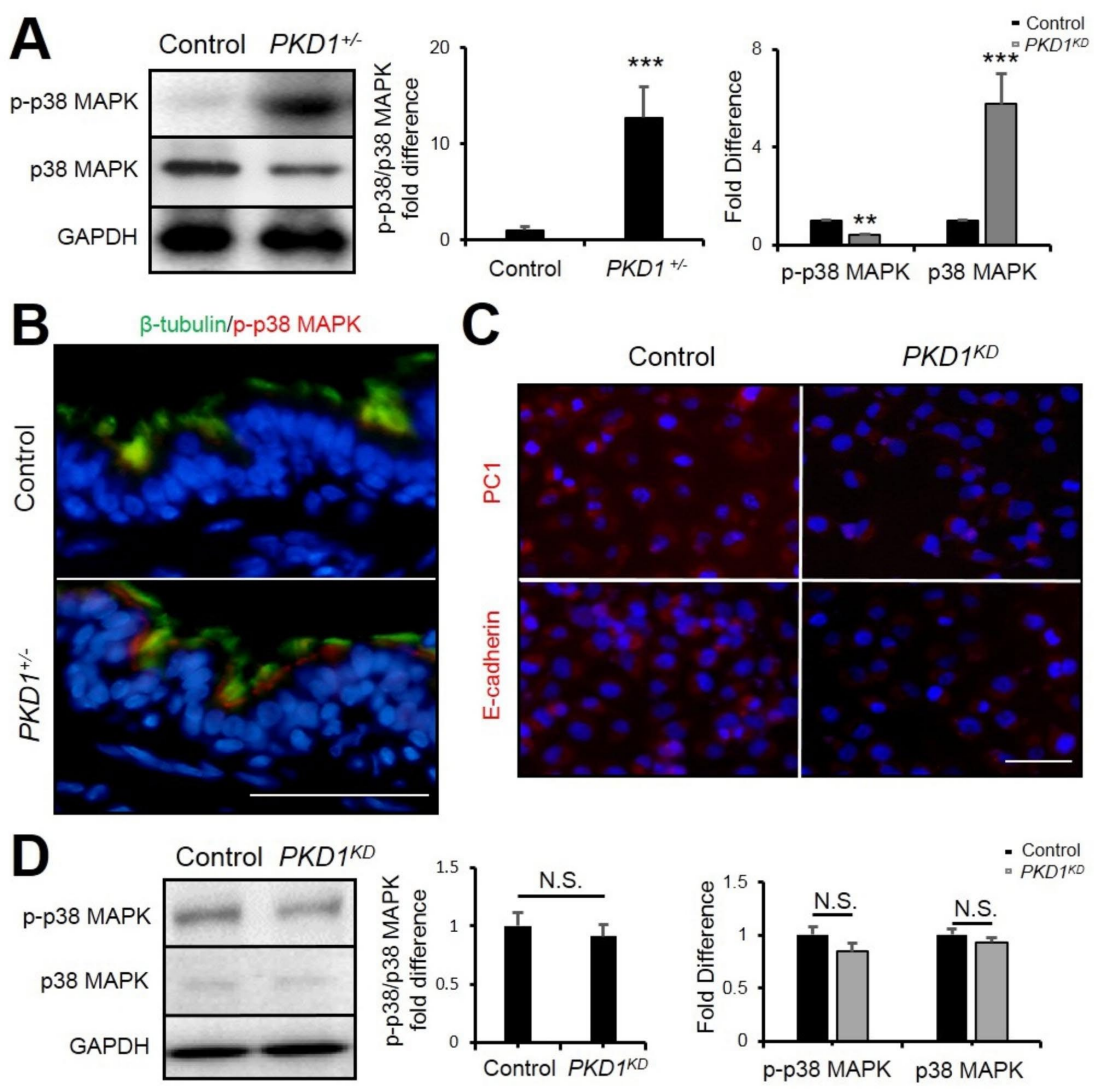
***PKD1*deficiency downregulated E-cadherin in lung epithelial cells. (A)** Representative Western blot and its quantification analyses of p-p38 MAPK and p38 MAPK in 2-year-old pig lungs. **(B)** Immunofluorescence staining of β-tubulin and p-p38 MAPK in 2 years old pig airways. Scale bar: 50 μm. **(C)** Immunofluorescence staining of PC1 and E-cadherin in Control and *PKD1*^*KD*^ H1299 cells. Scale bar: 100 μm. **(D)** Representative Western blot and its quantification analyses of p-p38 MAPK and p38 MAPK in Control and *PKD1*^*KD*^ H1299 cells. n = 3. The bars represent the mean ± SD; N.S. means no significant; **P <0.01; ***P<0.001.

Previous studies have shown that tobacco smoke downregulated E-cadherin expression in a p38 MAPK-dependent manner in bronchial airway epithelial cells [29]. p38 MAPK was also related to the skin and vascular endothelial cell barrier [30]. Thus, we examined the phosphorylation of p38 MAPK in the lungs from mutant and control pigs. In Western blot analysis, the phospho-p38 MAPK was significantly increased in the *PKD1*^*+/−*^ lungs (Fig. 4 A). Consistent with this result, the immunofluorescent staining analysis also showed a high level of p-p38 MAPK in the *PKD1*^*+/−*^ airway ciliated cells (Fig. 4B). To clarify whether p38 mediates *PKD1* deficiency induced downregulation of E-cadherin, we knocked down *PKD1* in a human lung epithelial cell line, H1299, by using the CRISPR-Cas13d system. Immunofluorescent staining and real-time PCR analyses showed that the expression of E-cadherin was markedly decreased in the *PKD1-*knockdown (*PKD1*^*KD*^) cells (Fig. 4 C & Supplemental Fig. 2). Interestingly, phosphorylation of p38 MAPK was not significantly altered in the *PKD1*^*KD*^ cells (Fig. 4D). These data indicate that p38 MAPK does not affect E-cadherin expression in *PKD1* deficiency.

### PKD1 deficiency affects airway smooth muscle cell layer in pig lungs

**Fig. 5:**
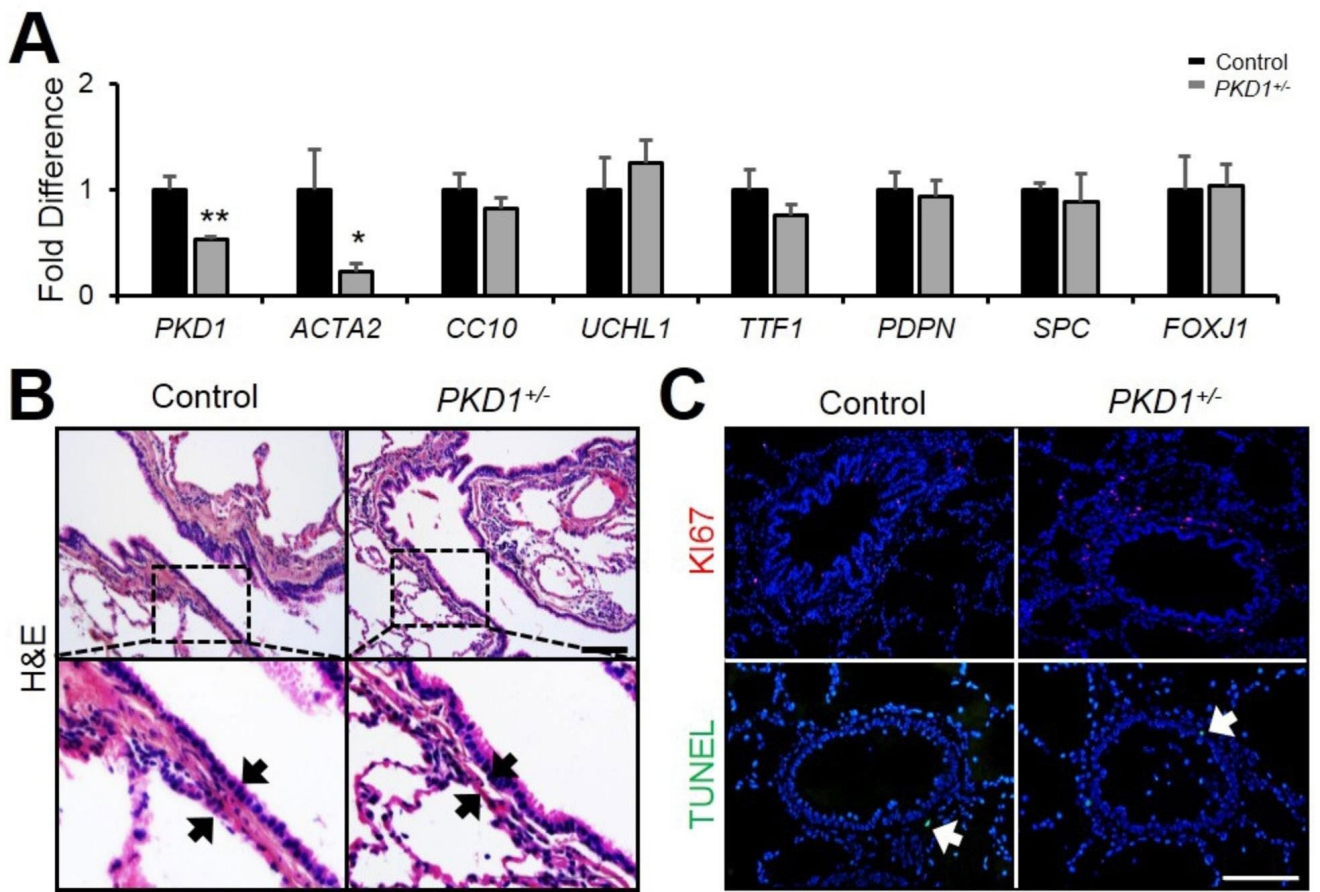
**Airway smooth muscle was altered in*PKD1***^***+/−***^**pigs. (A)** Real-time PCR of *PKD1* and cell marker genes expression in 2-year-old pig lungs. n = 3. **(B)** H&E staining of 2-year-old pig lungs. Scale bar: 100 μm. **(C)** TUNEL and KI67 staining of 2-year-old pig airways. Scale bar: 100 μm. The bars represent the mean ± SD; *P < 0.05; **P < 0.01.

To examine whether *PKD1* insufficiency in the mutant lungs affected epithelium and mesenchymal cells, real-time PCR analysis was performed to determine the expression of specific cell type markers. α-smooth muscle actin (*ACTA2*), Clara cell secretory protein (*CC10*), ubiquitin C-terminal hydrolase L1 (*UCHL1*), transcription termination factor 1 (*TTF1*), podoplanin (*PDPN*), and surfactant protein C (*SPC*) were used to detect mesenchymal smooth muscle cells and airway Clara cells, neuroendocrine cells, epithelial cells, alveolar type-I and type-II cells respectively. Compared with wild-type lungs, the expression of *ACTA2* was significantly reduced, whereas the expression of those epithelial cell markers was unchanged in the mutant lungs (Fig. 5 A). Consistent with this result, there were no differences observed in the expression of β-tubulin and CC10 between the *PKD1*^*+/−*^ and control lungs as shown by immunofluorescence staining (Supplementary Fig. 3). By H&E staining, the airway smooth muscle cell layer in the *PKD1*^*+/−*^ lungs was much thinner than in the control lungs (Fig. 5B), whereas the thickness of blood vascular walls were unchanged (Supplementary Fig. 4). To clarify whether the alteration of airway smooth muscle cells in the *PKD1*^*+/−*^ lungs was due to the increased cell apoptosis or decreased cell proliferation, TUNEL assay and anti-Ki67 staining were performed to measure cell death and proliferation respectively. The results showed no visible difference in cell apoptosis and cell proliferation between mutant and control lungs (Fig. 5 C).

### Airway smooth muscle cells undergoes phenotype switch in the PKD1^+/−^ pig lungs

**Fig. 6:**
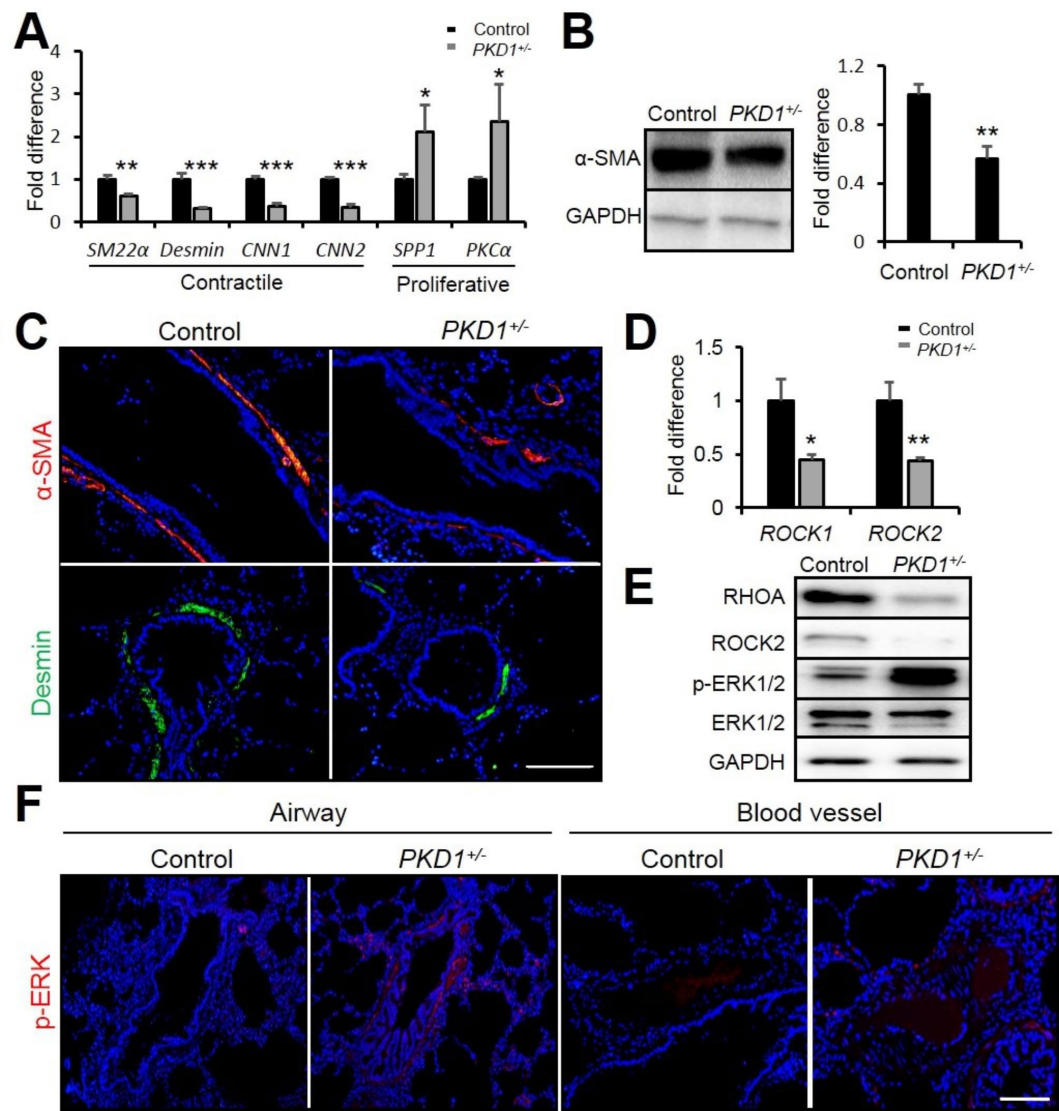
***PKD1*deficiency caused airway smooth muscle cell phenotype switch via ROCK and ERK signaling. (A)** Real-time PCR of genes related to smooth muscle phenotypes in 2-year-old pig lungs. n = 3. **(B)** Representative Western blot and its quantification analyses of α-SMA expression in 2-year-old pig lungs. n = 3. **(C)** Immunofluorescence staining of α-SMA and Desmin in 2-year-old pig airways. Scale bar: 100 μm. **(D)** Real-time PCR of *ROCK1* and *ROCK2* in 2-year-old pig lungs. n = 3. **(E)** Representative Western blot analyses of RHOA/ROCK and ERK signaling expression in 2-year-old pigs. n = 3. **(F)** Immunofluorescence staining of p-ERK in 2-year-old pig airways and blood vessels. Scale bar: 100 μm. The bars represent the mean ± SD; *P < 0.05; **P < 0.01; ***P < 0.001.

Airway smooth muscles (ASMs) play an important role in the process of airway remodeling in chronic airway disease [31]. ASMs exhibit phenotype plasticity through the reversible modulation and maturation of individual myocytes between contractile and proliferative phenotype [32]. Mature smooth muscle cells exhibited contractile term and dysfunction may lead to airway hyper-responsiveness and remodeling [33]. α-smooth muscle actin (ACTA2), transgelin (also known as SM22), Desmin, and Calponin are used as markers of the contractile phenotype [28], whereas high levels of CD44, protein kinase C (PKC), and osteopontin (OPN) are detected in the proliferative phenotype [34]. By real-time PCR analysis, the contractile phenotype markers (*SM22α*, *Desmin*, *CNN1* and *CNN2*) were reduced while the proliferative phenotype markers (*SPP1* and *PKCα*) were increased in the *PKD1*^*+/−*^ lungs (Fig. 6 A). In Western blot analysis, the level of α-SMA was significantly reduced in the *PKD1*^*+/−*^ lungs (Fig. 6B). Consistent with these results, the expression of α-SMA and Desmin in the *PKD1*^*+/−*^ lungs were shown to be decreased using immunofluorescence staining in the airway smooth muscle (Fig. 6 C), but not in the vessel walls (Supplementary Fig. 5). These data suggest that *PKD1* deficiency leads to airway smooth muscle cells phenotype switching from the contractile to the proliferation form, which might contribute to airway remodeling.

Previous studies have shown that ROCK signaling is involved in actin polymerization and promotes smooth muscle contraction [31]. Meanwhile, increased ERK phosphorylation was found to lead to proliferative phenotype [35]. By real-time PCR and Western blot analyses, the expressions of ROCK1 and ROCK2 showed to be decreased (Fig. 6D) whereas the phospho-ERK was upregulated in the *PKD1*^*+/−*^ lungs (Fig. 6E & Supplemental Fig. 6). Furthermore, the anti-p-ERK staining result showed that ERK phosphorylation was increased in airway smooth muscles but not in blood vessels in *PKD1*^*+/−*^ lungs (Fig. 6 F). These results suggest that the deficiency of *PKD1* inactivated ROCK signaling and promoted ERK signaling, which lead to airway smooth muscle phenotype switch in the *PKD1*^*+/−*^ lungs.

### PKD1 deficiency in pig lung alters extracellular matrix deposition

**Fig. 7:**
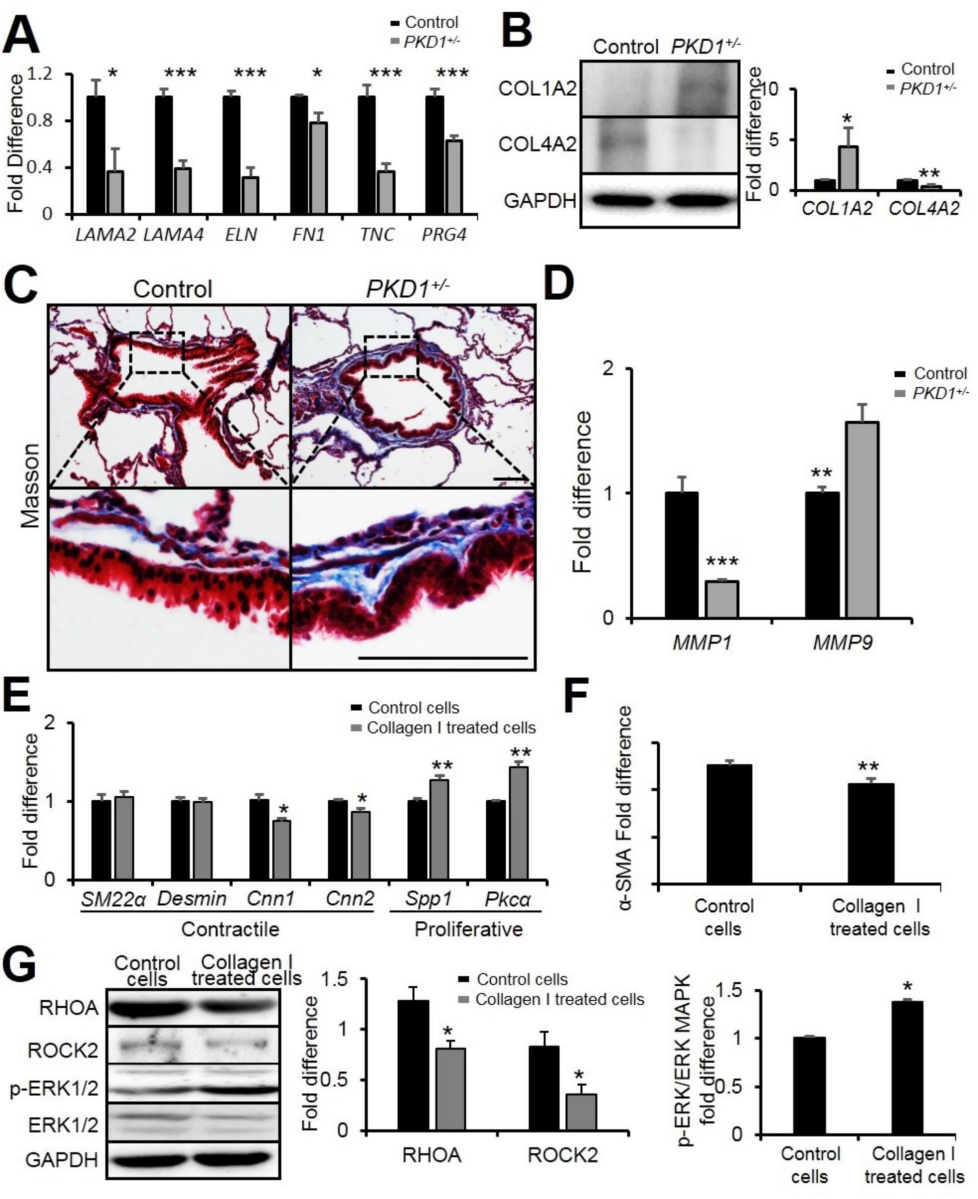
**Components of extracellular matrix in*PKD1***^***+/−***^**pig lungs were altered. (A)** Real-time PCR of genes related to extracellular matrix in 2-year-old pig lungs. **(B)** Representative Western blot analyses of COL1A2 and COL4A2 and their quantifications. **(C)** Masson staining of 2 years old pig airways. Scale bar: 100 μm. **(D)** Real-time PCR of *MMP1* and *MMP9*. n = 3. **(E&F)** Real-time PCR analyses of genes related to extracellular matrix and *α-SM* expression in control and Collagen I treated MRC5 cells. **(G)** Representative Western blot analyses of RHOA/ROCK and ERK signaling expression in control and Collagen I treated MRC5 cells. The bars represent the mean ± SD; *P < 0.05; **P < 0.01; ***P < 0.001.

Although *PKD1* was mostly expressed in the airway epithelial cilia cells (Fig. 1), the major effects by *PKD1* deficiency appear to occur in the airway smooth muscle layer (Fig. 5). Thus, the expression of extracellular matrix (ECM) components was analyzed to investigate the epithelial-mesenchyme interactions in the *PKD1*^*+/−*^ lungs. Several ECM proteins encoding genes were detected by real-time PCR. We found that the expression of laminin α2 (*LAMA2*), laminin α4 (*LAMA4*), elastin (*ELN*), fibronectin 1 (*FN1*), tenascin C (*TNC*), and proteoglycan 4 (*PRG4*) were downregulated in the *PKD1*^*+/−*^ lung (Fig. 7 A). ECM consists of a network of macromolecules including different types of collagens. Collagen I and Collagen IV regulate smooth muscle cell differentiation in various ways. Collagen IV enhances the expression of contractile genes, while collagen I reduces their expression [36]. By Western blot, the level of Collagen I showed upregulation whereas the expression of Collagen IV was decreased in *PKD1*^*+/−*^ lungs (Fig. 7B). In Masson staining analysis, abnormal collagen deposition was found around the *PKD1*^*+/−*^ airways (Fig. 7 C). In addition, *MMP1* and *MMP9*, whose translation products could degrade Collagen I and Collagen IV respectively, was decreased and increased correspondingly (Fig. 7D). These results indicate that *PKD1* deficiency inhibited MMP1 expression and in turn activated Collagen I. Meanwhile, the enhanced MMP9 expression in the mutant lungs resulted in Collagen IV reduction. The alteration of collagen I and IV further contributed to the phenotype modulation of airway smooth muscle cells in the *PKD1*^*+/−*^ lungs.

To confirm the airway smooth muscle cells phenotype switching from the contractile to the proliferation form was due to alteration of collagen I in *PKD1*^*+/−*^ lungs, MRC5 cells, human fetal lung fibroblast cells, were grown on collagen I coated culture plates. Consistent with previous results, the contractile phenotype markers (CNN1 and CNN2) were reduced while the proliferative phenotype markers (SPP1 and PKCα) were increased (Fig. 7E), and the expression of α-SMA was decreased in collagen I treated MRC5 cells (Fig. 7 F). By real-time PCR and Western blot analyses, the expressions of ROCK1 and ROCK2 showed to be decreased whereas the phospho-ERK was upregulated in the treated cells (Fig. 7G).

## Discussion

**Fig. 8:**
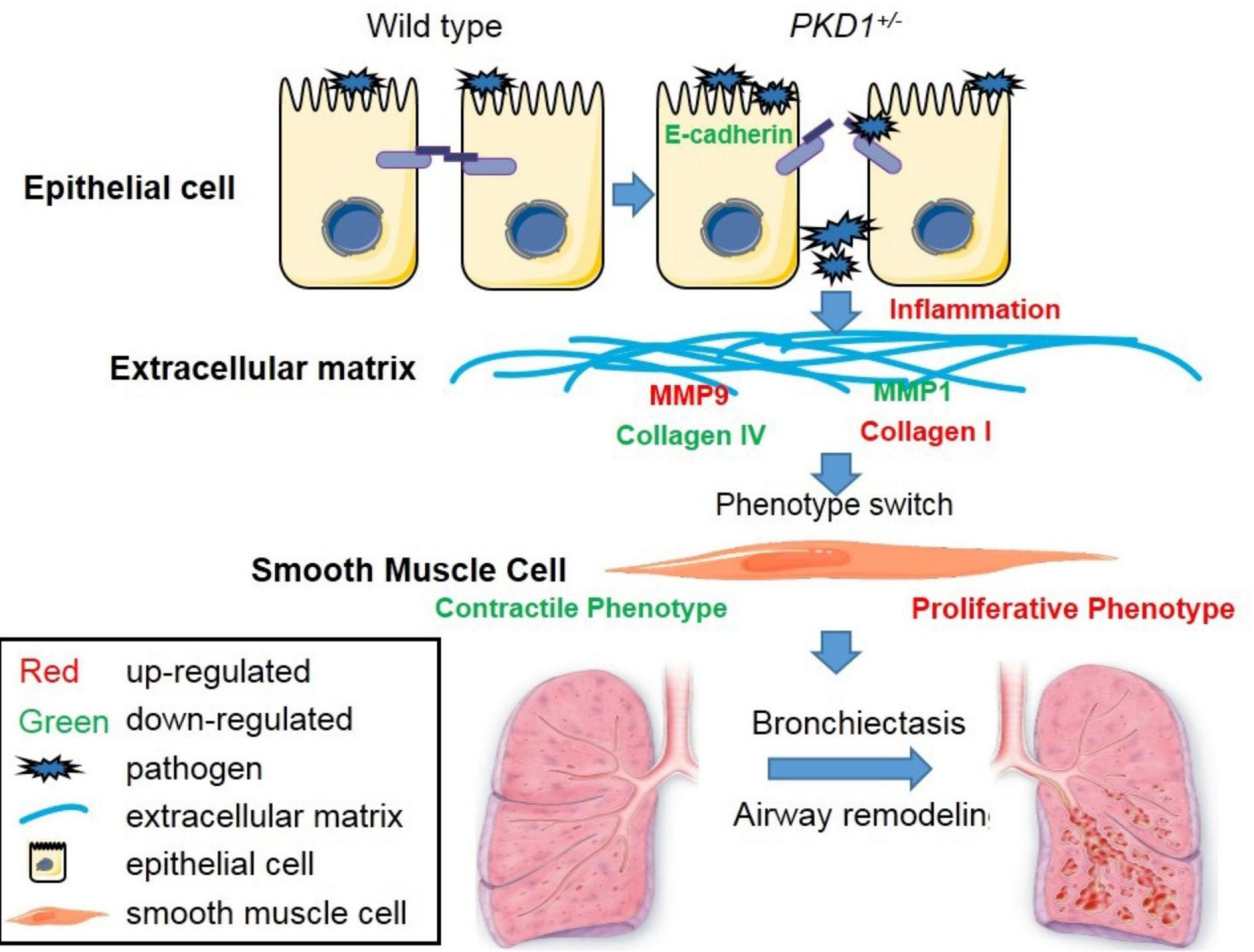
** A simplified model illustrating the role of*PKD1*in bronchiectasis.** In *PKD1*^*+/−*^ pig airway cells, *PKD1* deficiency caused E-cadherin downregulated, which leaded to epithelial barrier dysfunction. Along with the acceleration of harmful substance invasion and increase inflammation response, the components of airway extracellular matrix (MMP9, MMP1, Collagen IV and Collagen I) were altered, which in turn upregulated ERK signaling and inhibited ROCK signaling in airway smooth muscle cells, leading to the phenotype switch from contractile phenotype to proliferative phenotype. The increased dysfunctional airway smooth muscle resulted in the remodeling of airway and bronchiectasis

The purpose of this study was to investigate the mechanisms of bronchiectasis in ADPKD. ADPKD is the most common hereditary kidney disorder with an estimated prevalence of between 1/1000 and 1/2500 individuals. ADPKD is not only a renal disease but also a ciliopathy, and the patients with ADPKD also have bronchiectasis [9, 20]. *PKD1* deficient pigs were previously generated to simulate the progression of cyst formation in ADPKD patients [16]. In *PKD1*^*+/−*^ pig lungs, examination of bronchiectasis development revealed a sequential process of epithelial and mesenchymal interactions that lead to airway remodeling and bronchiectasis. Ablation of *PKD1* in the airway cilia resulted in interruption of expression of E-cadherin, which leads to dysfunction of the airway epithelial barrier, inflammation and accumulation of mucus. Due to the impaired pathogen clearance and extracellular matrix deposition in the *PKD1*^*+/−*^ lungs, airway smooth muscle cells undergo a switch from a contractile to a proliferative phenotype which drives airway remodeling and bronchiectasis formation (Fig. 8). These studies provide evidence that *PKD1*/E-cadherin signaling is critically involved in bronchiectasis by maintaining the epithelial barrier function and by regulating the balance of the airway smooth muscle cell contractile-proliferation phenotypes.

ADPKD is related to an increased prevalence of bronchiectasis in humans. By using CT scanning, bronchiectatic features, “tram line sign” and “signet ring sign”, were also observed in *PKD1*^*+/−*^ pig lungs. Bronchiectasis is a chronic lung disease with the presence of airway remodeling contributing to airway dilatation in patients [33]. Airway remodeling is the structural changes of airway wall and is usually found in chronic inflammatory airway diseases. Interestingly, increased inflammatory responses and excess mucus also occurred in the *PKD1*^*+/−*^ pig lungs. Thus, *PKD1*^*+/−*^ pig is a suitable animal model in which to investigate the underlying mechanism of bronchiectasis.

Cilia are classified as primary or motile. Primary cilia are non-motile and transiently present during the airway epithelial cell differentiation, and give way to motile cilia [5]. PC1 has been identified in both primary and motile cilia. The increased expression of PC1 during cilia differentiation suggests that PC1 is required for motile ciliogenesis [5]. Foxj1 is a marker of motile cilia, as the expression of Foxj1 was associated with a loss of primary cilia just before the appearance of motile cilia. We identified PC1 in all ciliated cells, marked by β-tubulin, in the airways of 2 years old pig, and the expression of *FOXJ1* was not altered in *PKD1*^*+/−*^ lungs. This observation suggests that PC1 deficiency did not affect motile cilia differentiation.

In the present study, our first aim was to determine whether the impaired mucociliary clearance in *PKD1*^*+/−*^ pig was due to the impact on airway epithelial cells by *PKD1* deficiency. Through breathing, the airway surface encounters an array of pathogens and toxic particulates. In response to these challenges, airway epithelial cells form a barrier to the outside world, and are at the frontline of mucosal immunity [37]. The epithelial barrier is comprised of airway surface liquids, mucus, and apical junctional complexes which consists of the apical TJ and underlying AJ that form between neighboring cells. TJ and AJ comprise two modes of cell-cell adhesion that provide different functions. TJ regulate paracellular pathway for the transportation of ions and solutes in-between cells, whereas AJ are important for initiation and maintenance of cell-cell contacts [38]. Our current study showed that *PKD1* deficiency in lung cilia cells reduced the expression of AJ components but not TJ families. In *PKD1*^*+/−*^ lungs, the decreased level of E-cadherin and β-catenin suggested that the barrier function is disrupted. Thus, it appears that the reduction of AJ components may lead to the loss of epithelial cell contacts and a leaky barrier that fail in mucociliary clearance.

Although there is growing evidence for disruption of epithelial barrier functions in airway disease, the pathophysiological significance and underlying mechanisms are currently unknown. Herein, by generating *PKD1*^*+/−*^ pigs and a *PKD1*^*KD*^ human lung epithelial cell line, we discovered that the deficiency of *PKD1* caused impaired airway epithelial barrier functions and bronchiectasis. Meanwhile, *PKD1* ablation in airway cilia cells also reduced E-cadherin levels. Earlier studies have reported that the E-cadherin protein directly interacted with PC1 in human kidney cells and pancreatic adenocarcinoma cells [39, 40]. Since E-cadherin is one of the most widely studied marker of adherens junction, the loss of E-cadherin in *PKD1*^*+/−*^ lungs may contribute to the impaired airway epithelial barrier function. There is evidence suggesting that p38 MAPK regulates E-cadherin expression in human bronchial epithelial cells [29]. Although p38 MAPK was upregulated in *PKD1*^*+/−*^ lungs, analysis of p38 MAPK in *PKD1*^*KD*^ cells showed that *PKD1* deficiency does not affect p38 MAPK. The possibility that a complex of PKD1 and p38 MAPK protein may participate in E-cadherin regulation or emergence of epithelial barrier functions was not addressed. However, this possibility is unlikely as PKD1 protein interacts with E-cadherin (CDH1) but not p38 MAPK by STRING (http://string-db.org/) analysis (Supplementary Fig. 7). Whether the increased p38 MAPK in *PKD1*^*+/−*^ lungs is a secondary effect of *PKD1* deficiency is a question that has not hitherto been addressed.

Airway smooth muscle (ASM) is now considered to play an active role in the regulation of airway remodeling in inflammatory airway diseases. The behavior of ASM cells in lung varies greatly depending on both intrinsic and extrinsic factors, and their phenotypic characteristics are dynamic. Mature ASM cells are normally characterized by a low proliferative index and expression of contractile markers. In inflammatory conditions, ASM cells adapt their functions by changing their phenotype from contractile to proliferative [41]. Since the ASM is physically associated with a bed of ECM, the phenotype of ASM is highly influenced by ECM [42]. The ECM consists of a complex network of interlacing macromolecules, which forms a supporting structure for the airway wall. The analyses in the present study showed the ASM phenotypes switched from contractile to proliferative in *PKD1*^*+/−*^ pig lungs, along with the expression of several ECM components were altered. Meanwhile, the expression of ROCK was reduced and the ERK phosphorylation was activated in *PKD1*^*+/−*^ pig lungs. Previous studies have described the roles of ROCK and ERK in ASM cell phenotype switch. By analyzing collagen I treated MRC5 cells, it confirmed that the phenotype switching and the alteration of ROCK and ERK signaling in *PKD1*^*+/−*^ pig lungs is due to the variations of ECM. In this study, the *PKD1* expression was almost undetectable in airway smooth muscle cells. Therefore, the ASM phenotype switch which drives bronchiectasis in *PKD1*^*+/−*^ lungs is most likely contributed by the deficiency of *PKD1* in airway cilia. Consistent with our findings, the work on selective inactivation of *PKD1* in SMCs showed no effects on contractility of arteries in mice [43].

In summary, the results of this study suggest that the *PKD1* deficiency inhibits E-cadherin expression and leads to dysfunction of cell barriers. This destruction increased the airway permeability and caused an aggravated inflammation response, which changed the components of the airway extracellular matrix, which in turn caused a phenotype modulation of airway smooth muscle cells via increased ERK signaling and downregulation of ROCK signaling and subsequently drove airway remodeling and development of bronchiectasis.

## Electronic supplementary material

Below is the link to the electronic supplementary material.

Supplementary Material 1

## Data Availability

The datasets during and/or analyzed during the current study available from the corresponding author on reasonable request.
