# Correction to: PKD1 deficiency induces Bronchiectasis in a porcine ADPKD model

**DOI:** 10.1186/s12931-022-02252-x

**Published:** 2022-12-22

**Authors:** Runming Wang, Wenya Li, Haiting Dai, Mingli Zhu, Lingyu Li, Guohui Si, Yilina Bai, Hanyu Wu, Xiaoxiang Hu, Yiming Xing

**Affiliations:** State Key Laboratory for Agrobiotechnology, College of Biological Sciences, China Agricultural University, Beijing, People’s Republic of China

## Correction to: Respiratory Research (2022) 23:292 https://doi.org/10.1186/s12931-022-02214-3

Following publication of the original article [1], the authors identified that the article note about equal contribution was missing.

It has been added in this correction and the original article has been updated.
