# Supplementary material for: PKD1 deficiency induces Bronchiectasis in a porcine ADPKD model
Source: Respir Res. 2022 Oct 29;23:292. doi: 10.1186/s12931-022-02214-3 (PMC9617414; doi:10.1186/s12931-022-02214-3)
Supplement: Supplementary file 1 — Supplementary Material 1 [file 12931_2022_2214_MOESM1_ESM.docx]

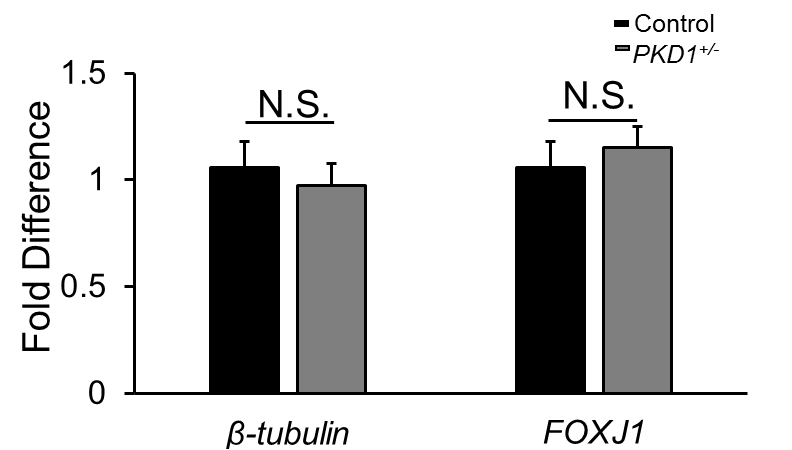


**Supplemental Figure 1. PC1 deficiency did not affect ciliated cell formation in *PKD1^+/-^* pig airways.**

Real-time PCR analyses of ciliated cell and motile cilia marker, β-tubulin and *FOXJ1* respectively, in 2-year-old Control and *PKD1^+/-^* pig lungs. n=3. The bars represent the mean ± SD; N.S. means no significant.


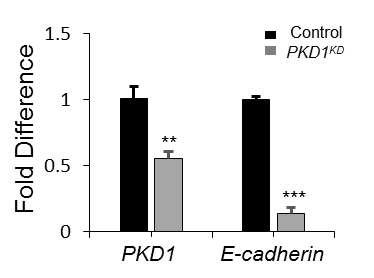


**Supplemental Figure 2. The expression of E-cadherin was decreased in *PKD1^KD^* cells.**

Real-time PCR analysis of *E-cadherin* expression. The bars represent the mean ± SD; ***P*<0.01; ****P*<0.001.


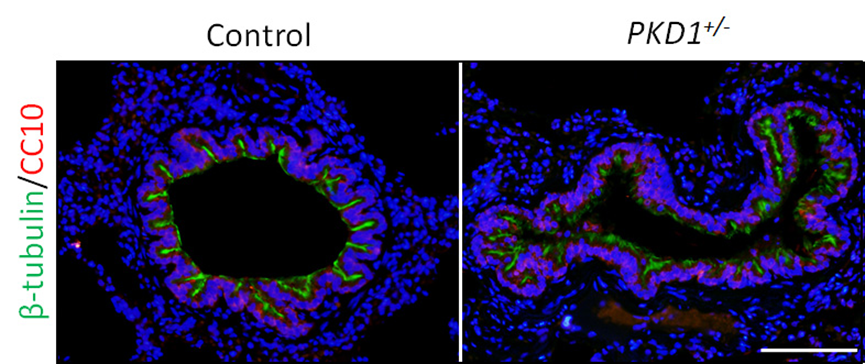


**Supplemental Figure 3. Club cell and ciliated cell were not altered in *PKD1* mutant 2-year-old pig lungs.**

Immunofluorescence staining of β-tubulin and CC10 in 2-year-old pig lungs. Scale bar: 100μm.


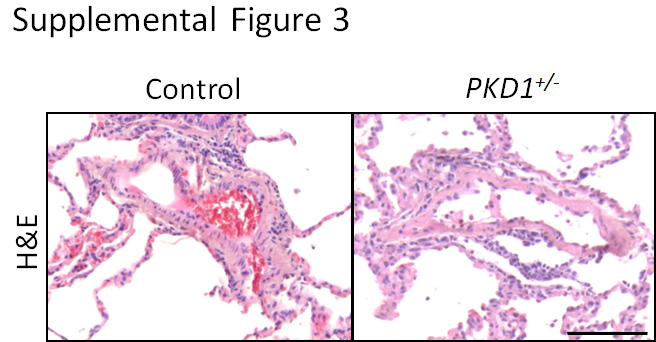


**Supplemental Figure 4. Vascular smooth muscle cells were not affected in *PKD1^+/-^* pig lung.**

H&E staining of vascular smooth muscle in 2-year-old pig lungs. Scale bar: 100μm.


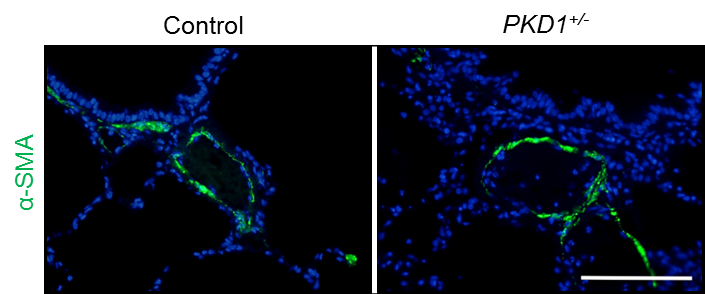


**Supplemental Figure 5. No phenotype switch was observed in *PKD1^+/-^* vascular smooth muscle cells.**

A. Immunofluorescence staining of α-SMA in 2-year-old pig lungs. B. Immunofluorescence staining of p-ERK in 2-year-old pig lungs. Scale bar: 100μm.


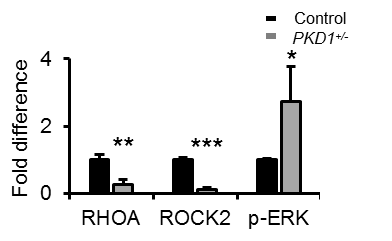


**Supplemental Figure 6.** **Quantifications of western blot analysis.**

The western blot analysis of RHOA\ ROCK2 and p-ERK in control and *PKD1^+/-^* lungs by Image J. The bars represent the mean ± SD; ***P*<0.01; ****P*<0.001.


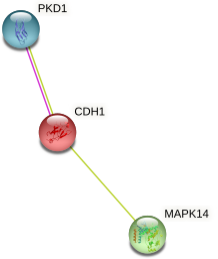


**Supplemental Figure 7. PKD1 protein interacts with E-cadherin, but not p38 MAPK.**

By STRING analysis, there is no interaction between PKD1 and p38 MAPK protein.


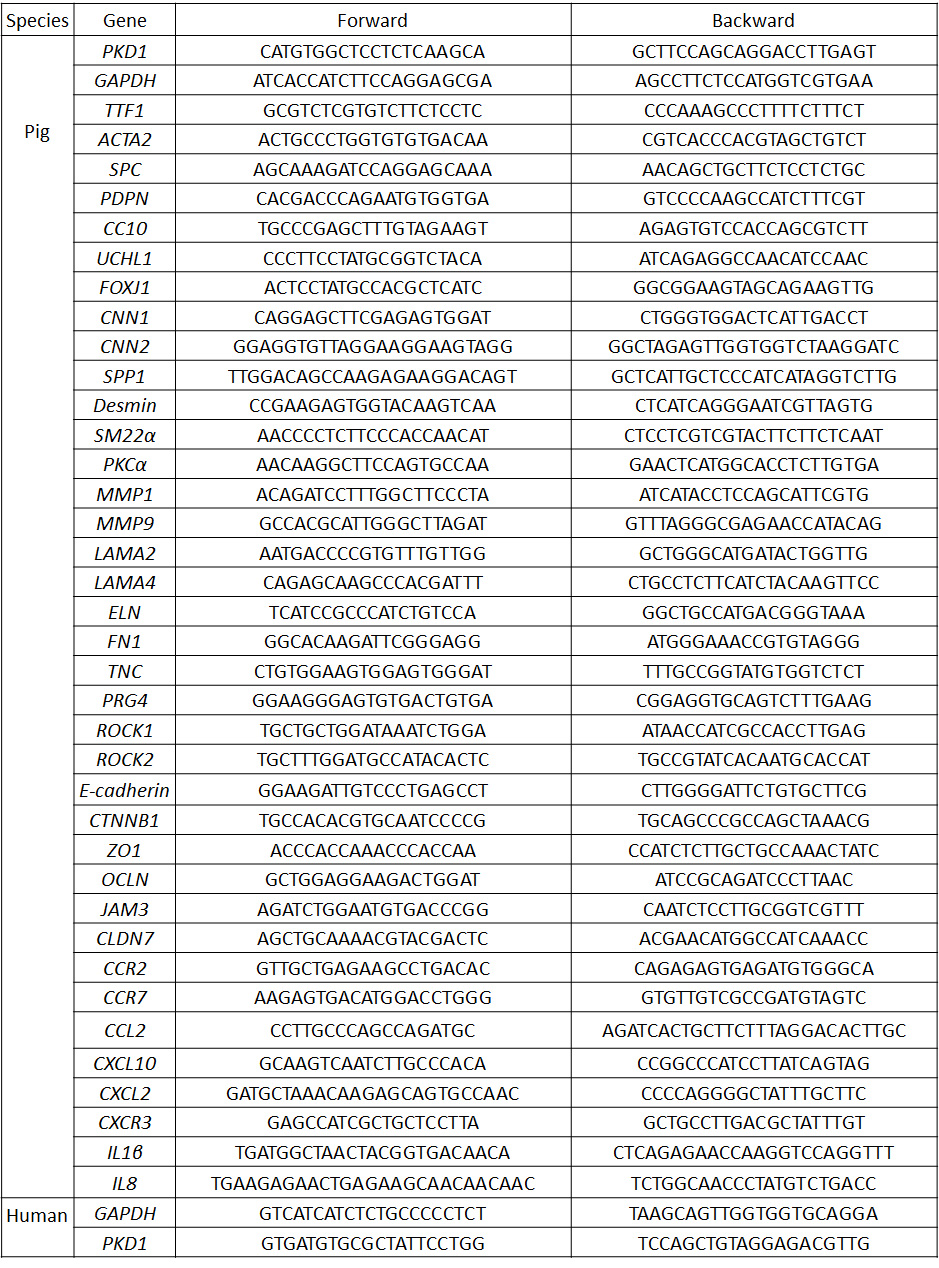


**Supplemental table 1. Primers used in this study.**


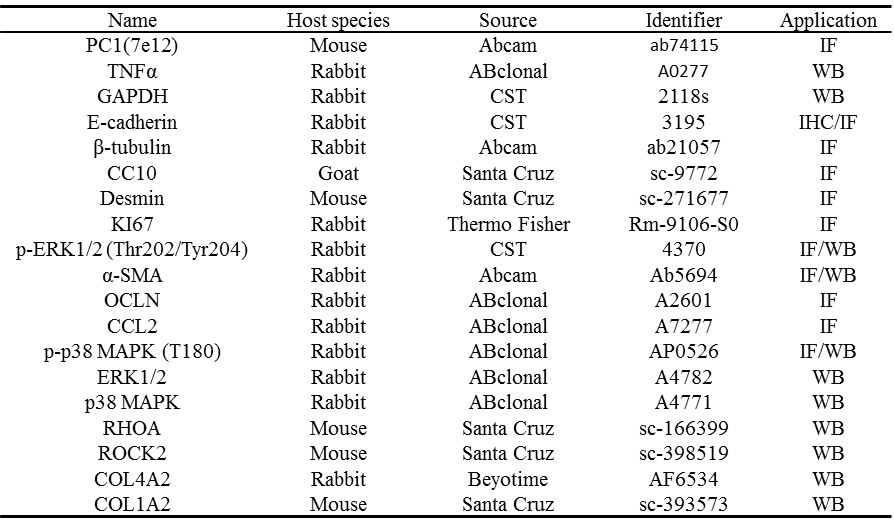


**Supplemental table 2. Antibodies used in this study.**
